# Open-Ended Coaxial Probe Technique for Dielectric Measurement of Biological Tissues: Challenges and Common Practices

**DOI:** 10.3390/diagnostics8020040

**Published:** 2018-06-05

**Authors:** Alessandra La Gioia, Emily Porter, Ilja Merunka, Atif Shahzad, Saqib Salahuddin, Marggie Jones, Martin O’Halloran

**Affiliations:** 1 Department of Electrical and Electronic Engineering, National University of Ireland Galway, Galway, Ireland; emily.porter@nuigalway.ie (E.P.); atifshahzad.m@gmail.com (A.S.); s.salah-ud-din1@nuigalway.ie (S.S.); marggie.jones@gmail.com (M.J.); martin.ohalloran@nuigalway.ie (M.O.); 2 Department of Electromagnetic Field, Czech Technical University in Prague, 166 27 Prague, Czech Republic; IljaMerunka@seznam.cz

**Keywords:** dielectric measurements, biological tissues, open-ended coaxial probe, equipment-related confounders, tissue-related confounders

## Abstract

Electromagnetic (EM) medical technologies are rapidly expanding worldwide for both diagnostics and therapeutics. As these technologies are low-cost and minimally invasive, they have been the focus of significant research efforts in recent years. Such technologies are often based on the assumption that there is a contrast in the dielectric properties of different tissue types or that the properties of particular tissues fall within a defined range. Thus, accurate knowledge of the dielectric properties of biological tissues is fundamental to EM medical technologies. Over the past decades, numerous studies were conducted to expand the dielectric repository of biological tissues. However, dielectric data is not yet available for every tissue type and at every temperature and frequency. For this reason, dielectric measurements may be performed by researchers who are not specialists in the acquisition of tissue dielectric properties. To this end, this paper reviews the tissue dielectric measurement process performed with an open-ended coaxial probe. Given the high number of factors, including equipment- and tissue-related confounders, that can increase the measurement uncertainty or introduce errors into the tissue dielectric data, this work discusses each step of the coaxial probe measurement procedure, highlighting common practices, challenges, and techniques for controlling and compensating for confounders.

## 1. Introduction

The interaction of electromagnetic (EM) fields with the human body is dependent on the inherent dielectric properties of each tissue. Based on these properties, electromagnetic waves are transmitted, absorbed, and reflected by biological tissues in different ratios. Accurate knowledge of these properties is crucial for dosimetry (safety) calculations and for medical diagnostic, monitoring, and therapeutic technologies.

The dielectric properties of tissues can be incorporated into highly accurate computational and physical models, and the generated preliminary data can be used to assess the technical risk, efficacy, and safety of the medical device or treatment. For instance, numerical models based on tissue dielectric parameters are used to calculate the specific absorption rate (SAR) in biological tissues. SAR levels are regularly calculated to validate the safety of many medical technologies, including magnetic resonance imaging (MRI) and implantable devices. Since SAR is a complex function of the dielectric properties of tissue, accurate knowledge of these properties are the foundation upon which SAR safety analysis is built [1,2]. Furthermore, accurate knowledge of the dielectric properties of biological tissue have prompted the development of a wide range of novel diagnostic and therapeutic technologies.

EM imaging ranges from the low-frequency Electrical Impedance Tomography (EIT) to higher-frequency Microwave Imaging (MWI). Both of these techniques rely on dielectric contrasts between organs or on contrasts between healthy and diseased, or inflamed, tissue. These imaging methods have gained significant academic and commercial interest, since both EIT and MWI are non-invasive and low-cost techniques [3,4,5,6,7]. While EIT is now established commercially for lung-function monitoring applications [8,9], MWI, similarly, has made considerable progress toward clinical usage in the past two decades as tissue dielectric properties enable the differentiation of benign and malignant tissues in breast cancer imaging [10,11,12,13,14], the monitoring of bladder volume in the treatment of enuresis and urinary incontinence [15,16], and the detection of stroke in intracranial imaging [17,18,19,20].

From a therapeutic perspective, knowledge of the relevant dielectric properties is used in the design and optimisation of hyperthermia (HT) [21,22,23], radiofrequency ablation (RFA) [24,25,26]**,** and microwave ablation (MWA) systems [27,28,29,30,31]. Hyperthermia consists of elevating the temperature of a diseased tissue to just above a normal physiological level in order to sensitise tumour cells, making the cancerous tissue more susceptible to chemotherapy and radiotherapy [32]. Targeted HT has been demonstrated to be particularly effective in the treatment of cervical cancer, breast cancer, cancers of the head and neck, and sarcoma in adults [21] and germ cell tumours in young children [23]. In EM-based hyperthermia systems, heating is achieved by coherently adding signals at the tumour location. In order to achieve coherent summing of the waves at the appropriate location, knowledge of the wave propagation speed is required, which depends on the dielectric properties of the tissues in the region. Similarly, radiofrequency ablation (RFA) and microwave ablation (MWA) are two treatments for liver, kidney, and lung cancer [33,34]. Both methods cause the direct necrosis of disease, and the relative high frequencies allow for good selectivity in terms of targeting the cancerous tissue, while protecting the surrounding healthy tissue [35]. Knowledge of the dielectric properties of tissues in the ablation region are factored into the design of ablation probes, where they are used to optimise the probe antenna efficiency and directivity, along with the size and shape of the ablation zone [36].

Thus, an accurate knowledge of the tissue dielectric properties not only has the potential to improve SAR estimates and reduce undesired tissue heating, particularly in newly developed RF-induction powered implantable sensors, but is also of key importance for the design of novel EM-based imaging and therapeutic technologies.

Due to the fast-paced development of novel, low-cost medical technologies and wearable devices, knowledge of new dielectric tissue data may be required. Thus, dielectric data may be acquired by researchers who are not specialists in the measurement of dielectric properties. For this reason, this paper reviews the most common measurement techniques for the acquisition of dielectric properties of biological tissues and references the most relevant dielectric studies in the literature.

There are several methods to measure the dielectric properties of biological tissues, including: The transmission line, cavity, tetrapolar (or multi electrode) probe, and open-ended coaxial probe techniques. Amongst these methods, the coaxial probe technique is the most commonly used [11,29,30,37,38,39,40,41,42,43,44]. Although the dielectric measurement process with an open-ended coaxial probe appears straightforward, different confounders can result in two types of errors in the measured data: Equipment-related (or system) and tissue-related errors. System errors relate to measurement equipment choice, measurement uncertainties, and measurement calibration and validation. Tissue-related errors are due to factors including: Temperature, probe-sample contact, probe-sample pressure, sample handling procedure, in vivo versus ex vivo experiments, tissue sample properties, and heterogeneity. Historically, equipment-related errors have been reduced with the development of a standard error correction calibration and good benchmarks have been defined to reduce or compensate for tissue-related errors. However, many tissue-related errors have yet to be investigated in detail. Both equipment- and tissue-related errors are addressed in this work. In particular, this paper focuses on the most common methods and best practices used to reduce or compensate for confounders affecting each step of the open-ended coaxial probe measurement process. Confounders are defined, here, as factors that affect the outcome (i.e., the measured dielectric properties) other than the intended cause (the actual tissue properties).

The remainder of the paper is organised as follows: Section 2 introduces the physical principles of the dielectric properties of biological tissues and summarises the most relevant works in the literature, highlighting the different aspects to consider in the process of tissue dielectric measurement. Section 3 describes the techniques used for dielectric measurement of biological tissues, and highlights why the open-ended coaxial probe method has, historically, been the most widely used for tissue measurements. In the following sections, the steps involved in an open-ended coaxial measurement are detailed. In Section 4, the standard calibration method is described and, in Section 5, the typical system validation procedure and the measurement uncertainty estimation are discussed. Tissue-related confounders are analysed in Section 6 and Section 7. Lastly, the paper concludes in Section 8, with a discussion proposing methods to refine the dielectric characterisation of human tissues and improve the interpretation of both historical and new dielectric datasets. It is hoped that this paper will be a useful reference text for those who are not experts in the field of dielectric data acquisition, but who are interested in using the resulting dielectric data or EM-based medical technologies that rely on this data.

## 2. Tissue Dielectric Properties: Background and Relevant Works 

This section provides the necessary theoretical background for understanding dielectric properties and their measurement. Firstly, dielectric properties are defined and their characteristics described. Then, a concise historical review of dielectric property measurements of tissues is detailed, highlighting the progress in the dielectric measurement of biological tissues to date. 

### 2.1. Basics of Dielectric Properties

The dielectric properties of biological tissues (and polar materials) are defined by the complex permittivity, ϵ(ω)*, which describes the interaction of the tissue with an external electric field. When an electric field is applied, a charge displacement in the tissue causes dielectric polarisation. The real and the imaginary terms of the complex permittivity are related by:

(1) $$\epsilon (\omega {)}^{*}=\epsilon^{\prime }(\omega )-j\;\epsilon^{\prime \prime }(\omega )=\epsilon^{\prime }(\omega )-j\frac{\sigma \left(\omega \right)\;}{\omega \epsilon_{0}},$$

where ω is the angular frequency. The real part of the complex permittivity, ϵ’, also called the “dielectric constant” or “relative permittivity”, expresses the ability of the tissue to store energy from an external electric field. The imaginary part of permittivity, ϵ’’, reflects the dissipative nature of the tissue, which absorbs the energy and partially converts it to heat. The conductivity, σ(ω), is linked to the imaginary part of the complex permittivity by the relationship defined in Equation (1). 

Equation (1) expresses the dependence of complex permittivity on the frequency of the applied external electric field. In particular, at specific frequencies, polarisation occurs and contributes to the tissue dielectric behaviour [45,46]. The dielectric spectrum of a tissue is characterized by three main dispersion regions, α, β, and γ, along with other minor dispersions, including the δ dispersion. These dispersion regions reflect the mechanisms occurring in various components of the biological material. Details regarding these biophysical mechanisms are thoroughly reported in [45,46]. 

Mathematical functions have been developed to model the dielectric behaviour of biological tissues and polar materials. These models are generally used to fit dielectric data, thus, reducing measurement data points to closed form equations and convenient graphical representations [11]. Dielectric models allow the calculation of the relative permittivity and conductivity values at any desired frequency within the range for which the relaxation equation is valid [47,48]. Importantly, these models allow for the dielectric properties of biological tissues to be easily incorporated into sophisticated computational models. 

The most common models used to describe the electrical behaviour of either aqueous electrolytic solutions or tissues are the: Debye, Cole-Cole, and Cole-Davidson models [49]. In general, the Debye, Cole-Cole, and Cole-Davidson models can be represented collectively by the Havriliak–Negami relaxation, which is an empirical modification of the Debye relaxation model, accounting for the asymmetry and broadness of the dielectric dispersion curve:

(2) $$\epsilon (\omega {)}^{*}=\epsilon_{\infty }+\frac{\epsilon_{s}-\epsilon_{\infty }}{{\left[1+{\left(j\omega \tau \right)}^{1-\alpha }\right]}^{\beta }}+\frac{\sigma_{s}}{j\omega \epsilon_{0}},$$

where ω is the angular frequency,$\;\epsilon_{\infty }\;$ is the permittivity at infinite frequencies due to electronic polarizability, ϵ_s_ is the static (low frequency) permittivity, σ_s_ is the static conductivity linked to charge movements, ϵ_0_ is the permittivity of the vacuum, α and β are empirical variables that account for the distribution of the relaxation time and the asymmetry of the relaxation time distribution, respectively, and τ is the characteristic relaxation time of the medium, which is the time necessary for the material molecules or dipoles to return to the relaxation state that was perturbed by the application of the electric field. When α = 0 and β = 1, Equation (2) corresponds to the Debye model. For 0 < α < 1 and β = 1, Equation (2) results in the Cole-Cole equation, which accounts for the distribution of the relaxation time. Lastly, for α = 0 and 0 < β < 1, Equation (2) corresponds to the Cole-Davidson equation, which is characterised by an asymmetrically broadened distribution of relaxation times [49]. While all of these models are used for fitting polar aqueous solutions, biological tissue data is generally fitted with the Debye and Cole-Cole models.

Equation (2) describes a single relaxation; however, if the dielectric behaviour of a material is analysed across a wide frequency range, all dielectric relaxations occurring over that frequency range must be taken into account and more poles (corresponding to the different relaxation times of the material) should be introduced to adequately describe the material. Biological tissues are generally described in terms of multiple Cole-Cole poles, which is a physics-based compact representation of wideband frequency-dependent dielectric properties [47].

### 2.2. Dielectric Property Studies in the Literature

Since the late 1940s, researchers have examined the dielectric properties of human and animal tissues across different frequency ranges, often using varied measurement procedures [50,51,52,53]. In the 1980s, the dielectric relaxation processes of biological tissues were further examined and modelled [45,46], and, increasingly, the open-ended coaxial line became the most common sensor for the acquisition of the dielectric properties of animal and human tissues [38,39,40,41,54,55,56,57,58]. The open-ended coaxial measurement technique was preferred to the transmission line, cavity perturbation, and tetrapolar probe methods, since the open-ended coaxial technique is non-destructive and allows for ex vivo and in vivo broadband measurements [39,59,60,61]. 

In the same decade, considerable progress was made on the measurement system and procedure, and several dielectric studies were conducted. Along with the dielectric characterisation of animal and human tissues [39,54,62,63], the tissue dielectric properties were analysed as a function of their physiological properties [45,55,64]. For instance, the dependence of the dielectric properties on tissue water content at microwave frequencies was analysed [56,65], the in vivo and ex vivo dielectric properties were compared [40], the difference between healthy and malignant tissues were examined [64,65], and the change in tissue dielectric properties post-mortem were reported [55].

A decade later, in 1996, Gabriel et al. published a comprehensive literature review reporting animal and human dielectric data across ten frequency decades, from 10 Hz to 20 GHz [66]. Dielectric data from a wide literature search was gathered and compared. Some inconsistencies were noted due to the use of different equipment and samples, and, therefore, Gabriel et al. sought to supplement these datasets with newly acquired data. Gabriel et al. completed in vivo and ex vivo animal and human tissue studies over a frequency range from 10 Hz to 20 GHz [42,47,67]. With this work, Gabriel et al. bridged gaps in the literature and consolidated the available dielectric data into one large dielectric repository. The experimental measurements were performed using three different techniques, depending on the acquisition frequency. To ensure quality, wherever possible, in vivo measurements on human patients were selected in preference to ex vivo or animal measurements. Where ex vivo/in vitro tissue was used, measurements were acquired as soon as possible after death. The data collected and measured by Gabriel et al. quickly became the generally accepted standard for dielectric properties of human tissues. This work was made publicly available on, firstly, the Federal Communications Commission (FCC) website [68] and on the Italian National Research Council (CNR) website, subsequently [69]. This broad availability allowed widespread use of the data among the scientific community and contributed to its diffusion.

In the subsequent years, dielectric measurement instrumentation and procedures were further improved. Specifically, the volume of the sample interrogated by the probe was investigated to accurately assign the acquired dielectric data to the actual tissue contributing to the dielectric measurement [70,71,72]. Based on the analysis of the probe sensing volume, precision probes were manufactured for localised dielectric spectroscopy of both low and high permittivity tissues [73].

In 2005, following an extensive measurement programme to measure the dielectric properties of several animal tissues, Peyman et al. described many measurement challenges related to the dielectric properties of biological tissues and corresponding methods to deal with them [43]. In 2006, Gabriel and Peyman reviewed tissue dielectric properties, with the aim of examining measurement uncertainties and their effect on existing dielectric measurements. The uncertainties were divided into random (“Type A”) and systematic (“Type B”), according to the guidelines defined by the National Institute of Standard and Technology (NIST) in 1994 [74,75].

In 2007, Lazebnik et al. examined the dielectric properties of breast tissue, with the aim of assessing the viability of using microwave imaging to detect early-stage breast cancer [11,76]. Through careful histological categorisation of each breast tissue sample, Lazebnik et al. found the breast to be dielectrically heterogeneous, and the dielectric contrast between fibroglandular tissue and cancerous tissue to be as little as 1.1:1 in the range between 0.5 GHz and 20 GHz. These findings were in conflict with almost all existing datasets, which had predicted considerably higher dielectric contrast (some as large as 10:1) [77,78]. The findings of Lazebnik et al. had a very significant impact in the community of researchers developing microwave breast imaging systems, since the data suggested that the dielectric contrast between healthy and cancerous tissue may be too low to clinically detect cancer using this technology. More recent works characterising healthy and cancerous breast tissue found a high variability in the properties across each tissue type and across patients, which complicates the dielectric differentiation between healthy and malignant tissue [12,58,79]. However, in Martellosio et al., a contrast in relative permittivity ranging from 1.1 to 5 was found between healthy and cancerous breast tissue across the range of 0.5–20 GHz [79], which is in broad agreement with the results of Lazebnik et al. [11,76].

In 2014, Sugitani et al. suggested that the inconsistency in the reported dielectric properties of breast tissue may be at least partially attributed to variations in the number of cells of each tissue type (e.g., fat or tumour) within a dielectric sample [12]. The findings in Sugitani et al. underscored the need to take into consideration tissue heterogeneity and histopathology within the sensing volume when completing dielectric studies. 

In order to define the sensing volume to account for histological analysis of heterogeneous biological tissues, Meaney et al. and Porter et al. examined the sensing volume of the common commercial dielectric probes and evaluated the dependence of the measured dielectric properties on the sample tissue composition [80,81,82,83].

Recently, numerous studies investigating the contrast in dielectric properties between healthy and malignant tissues have been conducted in order to improve the design of existing medical devices or to expand the clinical application of both imaging and therapeutic devices [10,37,84,85,86]. In particular, a number of works investigated the dependence of the dielectric properties of biological tissues on temperature for the optimisation of therapeutic technologies, such as RF/MW ablation [26,27,28,29,30,31,35]. 

To summarise, over the last three decades, notable progress has been made in the improvement of dielectric measurement equipment and in the refinement of the measurement protocol, aimed at further improving existing dielectric repositories. However, today, there is still a need for additional dielectric data to cover all tissue types, temperatures, and frequency ranges. This data provides the foundation for safety studies involving electromagnetic fields and for the design or optimisation of novel medical technologies. Therefore, in the next sections, the dielectric measurement procedure is discussed in detail and, along with each step of the procedure, the corresponding confounders that can introduce error into the results are discussed. Compensation techniques for mitigating the impact of confounders are also provided. 

## 3. Measurement Approaches

Different techniques have been used to measure the dielectric properties of tissue, including the transmission line and waveguide; open-ended coaxial probe; tetrapolar (or multi electrode) impedance; and perturbation cavity methods. In this section, an overview of each technique is provided, along with the known advantages and drawbacks of each. Then, the focus is on the most common method, the open-ended coaxial probe technique. This section underscores why the coaxial probe technique is the most used approach for dielectric tissue measurements. The state-of-the-art in modern open-ended coaxial probe measurement equipment is also presented.

### 3.1. Overview of Measurement Techniques

Among the measurement techniques used in previous dielectric studies, the most common methods are presented and briefly discussed in this subsection.

#### 3.1.1. Transmission Line

In transmission line measurement methods, a sample is placed in a coaxial line or, in the case of anisotropic tissue, in a rectangular waveguide so that the field polarisation may be varied. The transmission line is connected to two ports of a Vector Network Analyser (VNA) in order to acquire the scattering parameters (S11 and S21) [62,87], which are then converted into the complex permittivity (dielectric properties) of the tissue. The two most commonly used conversion methods are the Nicolson-Ross-Weir (NRW) method [88,89] and the NIST iterative conversion method [90,91]. The NRW method provides a direct calculation of permittivity from the complex reflection coefficient and the complex transmission coefficient obtained from the S-parameters [88,89,91,92]. Other common conversion methods are iterative and receive the initial guess from the NRW method or users’ input. The algorithm developed to implement the NIST iterative conversion method is reported in detail in Baker-Jarvis et al. [90].

The transmission line method allows measurement over a large frequency range, but only at low temperatures [87,93,94]. Waveguides are suitable for measuring larger samples (i.e., samples the size of the waveguide) at frequencies of up to 2.45 GHz, which is the frequency point normally used in microwave ablation. Smaller samples can be measured in the coaxial line, although this method also requires careful sample preparation in order to shape the sample to fit the line, and the method generally assumes that there are no air gaps in or around the sample and that the sample has smooth flat faces [95]. Thus, the transmission line method can be suitable for the measurements of biological fluids, but is unsuitable for in vivo measurements and not recommended for ex vivo measurements of semisolid or solid biological samples.

#### 3.1.2. Cavity Perturbation

The cavity perturbation method consists of a resonant cavity that resonates at specific frequencies. The tissue samples are inserted into the cavity and analysed by measuring the resonant frequency (f) and quality factor (Q), which are altered by inserting the tissue sample [94,95,96,97,98]. The tissue dielectric properties are then computed using the frequency, the Q-factor, and the sample volume. Details regarding the mathematical formulation to obtain the permittivity of the sample are reported by Campbell et al. [99]. However, the resonant frequency and quality factor are generally computed with a VNA. Since the maximum change in resonant frequency is achieved when a small perturbation occurs at the maximum intensity of the cavity mode, the cavity perturbation method requires a small sample [94,97]. Dielectric measurements performed using the cavity perturbation method can be accurate, but only provide dielectric data at a single frequency (in the upper microwave frequency range of 1–50 GHz). While the equipment needed for cavity perturbation measurements is readily available and cost-effective, the sample preparation is relatively complicated, requiring an excised tissue sample to be cut and moulded to a precise size and shape to fit into the cavity [95,97]. This process may introduce air pockets within the sample or between the sample and the cavity, loss of fluid in the tissue (which would affect its properties), and an increase in density from pushing the tissue into the cavity (which could also affect its properties) [97]. Due to the required sample size and, thus, sample preparation, biological tissue measurements with the cavity perturbation method are highly challenging.

#### 3.1.3. Tetrapolar Impedance

Unlike the previous two techniques, the tetrapolar (or multi electrode) impedance method is non-destructive and allows for in vivo tissue measurements. The tetrapolar probe is composed of four electrodes: Two of the electrodes are driven with a current source and the other two electrodes are used for voltage measurements. The two electrode pairs are used for impedance measurements, avoiding interference from effects related to the electrode-tissue interface [100,101]. The tissue dielectric properties are easily evaluated from the measured impedance with knowledge of the sample dimensions. Although the tetrapolar probe method does not require tissue processing and is very sensitive to tissue anisotropies [19,101], it is only suitable for specific low frequencies (in the range of 10^−6^–100 MHz) [101]. For the tetrapolar probe technique, the electrode configuration should vary according to the interrogated tissue. In order to increase the number of applications, tetrapolar probes may be replaced by spring-loaded multi electrode probes [102]. The multiple surface electrodes permits the setting of a current pattern so that the resulting measured voltage is more sensitive to a local area and less sensitive to other regions. Multi electrode probes can provide improved bioimpedance and anisotropy measurements [102].

#### 3.1.4. Open-ended Coaxial Probe

**Figure 1:**
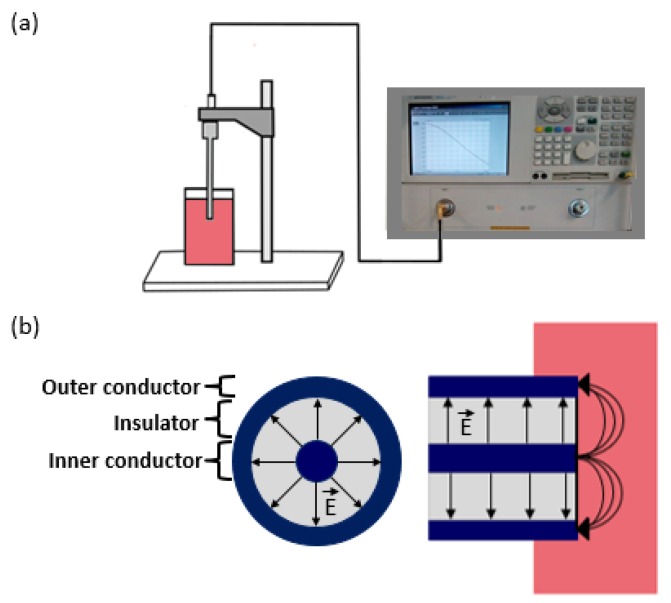
Open-ended coaxial probe technique: (**a**) Schematised measurement set-up, including the Vector Network Analyser (on the right), the cable connecting one port of the VNA to the coaxial probe, the probe bracket, and the liquid sample being measured; (**b**) top and side cross-sections of the coaxial probe, with electric field orientation indicated.

The coaxial probe technique does not suffer from many of the disadvantages associated with the techniques described above. The open-ended coaxial probe consists of a truncated section of a transmission line. The electromagnetic field propagates along the coaxial line and reflection occurs when the electromagnetic field encounters an impedance mismatch between the probe and the tissue sample. The open-ended coaxial probe measurement set-up and the probe cross-section are schematised in Figure 1. The reflected signals at different frequencies are measured and then converted into complex permittivity values.

Different methods have been developed to convert the measured reflection coefficient to permittivity [60,103,104,105,106,107]. However, today, this process is generally done automatically by software embedded in the VNA [108]. Therefore, details on the various methods are not discussed in detail here, but more information can be found in [103,104,105,106,107,109,110,111]. 

The open-ended coaxial probe has become the most commonly used method to measure the dielectric properties of tissues for several reasons: The method is simple; sample handling is minimal and non-destructive; and both ex vivo and in vivo measurements over a broad frequency range are possible [39,42,43,72,94]. However, the open-ended coaxial method assumes a homogeneous sample that is in good contact with the probe; therefore, air bubbles and uneven sample surfaces can result in inaccurate measurements [95], and heterogeneous samples present a particular challenge. There are also limits to the magnitudes of material properties that can be measured reliably [95]. The limits of what can be measured depend on a number of factors, including the probe design and materials (and, therefore, its impedance), precision of the probe fabrication procedure, calibration procedure (standards used), and the capabilities of the measurement device (i.e., the VNA). Furthermore, what is classified as a “reliable measurement” depends on the experiment and the required accuracy. Although theoretical limits of the measurement set up can be estimated analytically, they are generally estimated experimentally by measuring materials (usually liquids) with different extreme values of relative permittivity and conductivity. Then, the accuracy of the measurement can be estimated in different ranges of complex permittivity and it can be determined if the accuracy is appropriate for the experiment of interest.

Overall, many challenges associated with tissue dielectric property measurements may arise in each of the above measurement techniques, for example, issues related to temperature change and tissue heterogeneity. Since the coaxial probe technique is by far the most commonly used method for tissue measurements [10,11,12,39,40,41,42,44,59,60,62,112,113], it will be examined in more detail in the subsequent sections. 

### 3.2. Evolution of the Coaxial Probe Design and Fabrication

In the 1980s and 1990s, researchers conducting studies on dielectric measurements of biological tissues focused on probe design and fabrication, system development, and systemic error correction techniques [39,40,60,61,62,73]. The majority of the custom probes were fabricated from 50 Ω semi-rigid coaxial cables [39,40,60,61,62,94]. Probes were customised depending on the type and size of the tissue sample to be investigated and on the desired frequency range of the dielectric properties study. 

Several custom-made probes were made of metal and Teflon [39,40,61,62]. Burdette et al. used a 2.1 mm diameter probe to perform in vivo and ex vivo measurement on animal tissue over the frequency range 0.1–10 GHz. This probe had a flange (i.e., a ground plane) to contain the electromagnetic field at the tip [39]. Kraszewski et al. performed in vivo animal measurements over the frequencies 0.1–12 GHz using a Teflon-filled metal probe with a 3.2 mm external diameter [40]. Gabriel et al. used two Teflon-filled metal probes for in vivo and ex vivo animal studies in order to acquire tissue dielectric properties at both low and high frequencies [42]. The probe used in the low frequency range (10^−4^–200 MHz) had an external diameter of about 10 mm and the smaller probe, used for dielectric measurements at the frequency range between (0.2–20 GHz), had an external diameter of 2.9 mm [109]. Larger probes require a larger sample size due to the increased sensing volume (i.e., the region of the tissue that is interrogated by the electric field of the probe). In both Burdette et al. and Gabriel et al., the probe tips of the inner and outer conductors were plated with an inert metal, such as gold and platinum, to modify the effect of electrode polarisation, which is a manifestation of chemical reactions between the probe and the electrolytes (water molecules and hydrated ions) in the tissue [39,42]. Specifically, this plating process shifts the electrode polarisation, normally occurring at low frequencies, to even lower frequencies [39,46,109]. Popovic et al. reported that Teflon-filled copper probes, usually used for broadband reflection coefficient measurements, can cause inaccurate measurements because the probe aperture deteriorates easily and mechanical flaws can occur. The effects of small mechanical imperfections at the probe tip were quantified by the measured reflection coefficient and it was found that mechanical flaws at the probe tip can impact measurements by altering the reflection coefficient by up to 30% [114]. Notably, Teflon-filled copper probes do not meet bio-compatibility requirements nor can they be autoclaved (steam sterilised), both of which are required for safe in vivo measurements on human patients [73]. 

More recently, borosilicate glass-filled, stainless-steel, open-ended coaxial probes were designed and fabricated [73,115]. The use of thermally constant and matched, inert, refractory materials made the probe biocompatible and suitable for high-temperature sterilisation [73]. 

Over the last decade, a growing number of dielectric studies have been conducted using commercial probes [10,44,84,86]. Modern commercial probes are accurate [115], yet require specific sample dimensions and characteristics. In particular, Keysight probes, including the slim form probe, the performance probe, and the high temperature probe, have been used in most of the recent tissue dielectric studies [12,44,79,86,116]. Out of these, the slim form probe is a common choice for tissue measurements due to its small diameter and the fact that it can be steam-sterilised and, thus, used in vivo. The tissue dielectric measurements performed using these commercial probes are summarised in Table 1. 

As the open-ended coaxial probe has been demonstrated to be the most applicable to measuring the dielectric properties of biological tissues, the remainder of this work will focus on the dielectric measurement process using this probe, from system calibration to biological sample preparation and analysis. In the next section, the calibration procedure for open-ended coaxial probes is discussed.

## 4. Calibration and Confounders

A standard calibration procedure, involving both the coaxial probe and the VNA, must be performed before recording dielectric measurements [40,60,62,117]. In this section, a description of the calibration process is provided, followed by an in-depth analysis of the related confounders.

### 4.1. Standard Calibration

In general, coaxial probe measurements use a three load standard calibration procedure for one-port error correction. Any three different standard materials can be used for calibration, as long as the dielectric properties of those standards are well known [117,118,119]. The choice of standard materials to use may be based on ease of use, availability, or similarity to the materials under test [94,117]. The three most common standards used for coaxial probe calibration are: Open circuit, short circuit, and a broadband load [114,115]. We note that the use of the term “broadband load” here does not indicate a perfectly matched load, but rather, the broadband load can be any liquid with known dielectric properties. The calibration is performed at the reference plane of the probe, while the probe is connected to the VNA. The probe may be connected directly to the VNA or through a phase-stable cable. The calibration procedure aims to find a relation between the measured complex reflection coefficient and the expected one. This procedure allows for all post-calibration measurement data to be corrected [120]. If performed correctly, a good calibration procedure results in reliable measurements. The quality of the calibration depends on the accuracy in the measurements of the three standards and on the level of control over the factors that can affect the process. In the following subsection a list of the calibration steps required to reduce the confounders is reported. In addition, the confounders and methods for their compensation are summarised in Table 2.

### 4.2. Calibration Procedure and Confounders

#### 4.2.1. Equipment Set-Up and Confounders

Before performing the calibration, environmental parameters, such as temperature, pressure, and humidity, should be controlled or monitored [122,127] because environmental changes may impact measurement results [74]. Furthermore, system components should be checked [39], the probe tip cleaned and verified by visual inspection [29,41,42,43], and the cable (if not phase-stable) fixed in place [29,44,86] as imperfect connections [39], probe contamination [27,37,39,121], and cable movement [10,27,43,44,76,80,86,95,115] can all result in a poor calibration and, thus, unreliable measurements.

#### 4.2.2. Signal Settings and Confounders

Prior to calibration, the frequency range needs to be selected based on the planned experiment. Subsequently, the number of acquisition frequency points must be defined. Frequency points may be equidistant according to a linear or a logarithmic scale. The use of a logarithmic scale can be advantageous when data is acquired over a larger frequency range as there will be more points taken at the frequency points where the largest change in dielectric properties occurs (due to dispersions) [128]. The signal power and measurement bandwidth must also be selected in the VNA software. The number of points and bandwidth requires a trade-off between the measurement accuracy and speed of data collection.

#### 4.2.3. Measurement of the Three Standards and Confounders

Once the measurement settings are selected, the calibration measurements of the open-circuit, short-circuit, and broadband load can be performed. The common errors and confounders likely to occur during the calibration process are highlighted in Table 2, along with the recommended correction and compensation techniques. As noted in the table, while performing calibration, (when using modern VNAs) visualisation of the complex impedance on the VNA Smith chart is key to identifying the unwanted presence of particles at the probe tip and confirming the quality of the open or short circuit [56,95]. In particular, having a good quality short circuit is vital to a successful calibration [94]. Therefore, proper contact between the short and the probe must be ensured prior to completing the calibration. Other than this, the open and short measurements are relatively straightforward and do not require any additional consideration. In the case that the VNA does not allow visualisation of the Smith chart during calibration, the quality of the calibration can then be verified by performing the validation procedure, as described in Section 5.

Conversely, several confounders can introduce error into the load measurement. Different liquids have been examined as potential load materials. The permittivity of the standard liquid should be selected such that the complex impedance of the load is considerably different from the other two standards [129]. The most typical liquid used as a load is deionised (DI) water [12,27,80,86,116,130]. Polar liquids (for example, ethanol, methanol, and saline) also meet the requirements [129] and exhibit high conductivity and permittivity as a function of frequency. Nyshadham et al. examined the effect of the uncertainty of the models of different standard materials on the uncertainty of the measured permittivity [117]. In this study, different liquids (having different models) were used for calibration and it was verified that DI water has smaller uncertainties in the Debye model than that of other standard liquids (in other words, the dielectric properties of deionised water are the most well-known and well-characterised) [117]. Indeed, the accuracy of the model represents one of the confounders affecting the calibration procedure and the uncertainty of the measured permittivity. Specifically, a quantitative analysis that examined the impact of errors in the model of one of the calibration standards (in this case, acetone) found that model errors of 2% induced a similar magnitude of error into the measured relative permittivity [131]. However, despite the impact of model uncertainties, the best calibration material depends on the measurement scenario as the uncertainty will be lower for materials measured with properties similar to those of the calibration material. 

##### Temperature of the Liquid

During the calibration process, the temperature of the load liquid needs to be maintained and monitored, since dielectric parameters are temperature-dependent [43,94,124,125]. The permittivity of liquids vary by up to 2.2% per degree Celsius [125]. The measurement of deionised water, or any standard liquid, as a calibration load may be performed at room temperature or at any fixed temperature. In the first case, the liquid temperature can be monitored using a thermometer [95]. In the second case, the temperature may be maintained using a water bath [29,43,44,121]. In addition, if the temperature of the liquid is different from the temperature of the probe, it is recommended to wait for the temperature to stabilise before proceeding with the measurement. We note that this information on the liquid temperature also applies to the liquid used in the validation step.

##### Other Confounders in the Liquid Measurement

Aside from the liquid temperature and model accuracy, other confounders, such as liquid contamination [43], air bubbles between the probe and the liquid [48,71,95,126], and probe position in the liquid-filled beaker [71], have been investigated. These confounders affect the load liquid used during calibration and the liquid used in the validation step equally—indeed, the same types of reference liquids can be used either for calibration or for validation. 

In order to avoid any impurity in the water, the beaker filled with liquid should be kept closed [43]. The presence of air bubbles between the probe tip and the standard liquid can result in deviations in the dielectric measurement data by up to 20% due to the fact that the material within the sensing region is then a mixture of air and liquid [126]. A transparent beaker is recommended so that air bubbles can clearly be seen. If bubbles are present, they need to be removed prior to measurement. This may be completed by gently tapping the probe tip on the bottom of the beaker, or lowering the beaker away from the liquid and then re-immersing it on an angle [95]. A soft brush (non-metallic, to avoid scratches) may also be used to remove any bubbles without having to move the probe or the beaker. In addition, the probe should be immersed in the liquid and positioned in the beaker such that the liquid is the only material within the probe sensing volume. Accurate positioning avoids undesirable reflections from the beaker walls. Hagl et al. provided a process for finding the minimum distance between the probe and the beaker sides according to the probe size; these distances also depend on the properties of the liquid material in the beaker and the frequency range of interest [71].

### 4.3. Confounders Introduced in the System after Calibration

Following the calibration procedure, two additional system confounders can introduce errors in dielectric measurements: VNA drift over time and cable movement, although the movement of a phase-stable cable should not compromise the performance of the system [10,27,43,44,76,80,86,115]. The system drift should be characterised and taken into account in the measured dielectric data [43,44]. This factor can be quantified by taking several measurements on a standard liquid at defined time instants in the period after calibration [43]. When a cable that is not phase-stable is moved, given the difficulty in precisely characterising the systematic error introduced by the cable movement, a new calibration is required. However, low loss and phase-stable cables should be used to minimise the impact of the error of the cable stability on the results [71,76,94]. In some works, the cable was fixed in place (using adhesive tape) to limit the effect of the cable movement in the dielectric data [29,44,86]. An alternative approach may be to replace the cable with a right-angle connector, when the rigid set-up does not overly restrict dielectric data acquisition [128].

After each calibration, it is good practice to first confirm proper calibration by re-measuring one of the calibration standards, commonly the short [44]. Note that re-measuring the properties of materials used during calibration does not guarantee that the system is functioning error-free, it just indicates that the calibration error-correction algorithms were successfully applied. Thus, a measurement of a known liquid, other than the one used in calibration, is also required in order to validate the accuracy of the calibration. Details about the validation procedure and the measurement uncertainty calculation are discussed in the next section.

## 5. Validation and Measurement Uncertainty

The validation procedure consists of measuring the dielectric properties of a known reference liquid. To ensure that the measurements are accurate in materials with different properties, the validation material should not be the one used during the calibration (i.e., typically not deionised water). Validation enables determination of the quality of the calibration and the monitoring of systematic errors [43,44,75], such as VNA drift and noise due to cable movement [43]. Thus, it is good practice to perform validation immediately following calibration [39,43,71,121,132] and after acquiring a set of tissue dielectric data [76]. The validation should also be completed whenever anomalies are observed in the dielectric data of the investigated material in order to isolate the source of error. For instance, if the same anomalies are observed in the reference liquid dielectric trace, the error is due to changes in the system and a new calibration is needed; if the anomalies are not evident in the liquid trace, the error is sample-related and further investigation is needed to identify the source of the error.

During the validation procedure, monitoring or controlling the temperature of the liquid during the validation process is required, since the dielectric properties of reference liquids are temperature- and frequency-dependent [43,44,132]. 

Although system validation is a simple procedure, several confounders can introduce errors in the process. The factors that affect the validation quality are similar to those present in the load measurement during the calibration procedure. Thus, details regarding confounders in the liquid dielectric measurement and how they are addressed can be found in the previous section. 

In this section, after describing the most common validation liquids, the role of the validation procedure in the calculation of the uncertainty of dielectric data is detailed.

### 5.1. Validation Liquids: Models, and Their Advantages and Disadvantages

Alcohols and saline are the most common polar reference liquids [39,71,75,76,125,133]. Polar solutions are particularly suitable as validation liquids because they have comparatively high relative permittivity and high dielectric loss at radio and microwave frequencies. Both the relative permittivity and conductivity have a strong frequency dependence, which is a feature of the pronounced molecular dielectric relaxation behaviour [94]. Liquids, in general, are selected for validation purposes as they are homogeneous and are free of many of the confounders affecting solids or semi-solids (e.g., incorrect probe-sample contact, inconsistent probe-sample pressure).

#### 5.1.1. Alcohols

Methanol, ethanol, ethanediol, and butanol are the types of alcohols generally used to characterise the system and calculate the uncertainty in the dielectric measurements [44,71,72,76,117,125,132] prior to tissue measurements. Methanol, ethanol, and butanol, in particular, are used as standard liquids because they represent the high, intermediate, and low dielectric property values, respectively, within the range of those expected for human breast tissues at microwave frequencies [71,72,76,132]. They also have well-established permittivity models [72,76,125,132]. Ethanediol, which has also been modelled in the microwave frequency range [44,132,133,134], has a static permittivity about half that of pure water [134]. Standard methods for obtaining the known dielectric property values for each of these alcohols have been detailed thoroughly [132].

Although alcohols present properties similar to those of biological tissues at microwave frequencies (0.5–20 GHz), there are some constraints that must be taken into account when using them as reference liquids. For instance, the alcohol models are accurate in restricted frequency ranges and at discrete temperatures only [117,124,132,134,135]. Furthermore, the dielectric properties of alcohols can change during storage and handling. For example, methanol has very low vapour pressure and evaporates rapidly. This can contribute to a decrease in the liquid temperature and, consequently, to a dielectric property change over the course of just a few minutes when exposed to air [72,132]. In order to minimise these effects, the dielectric properties of methanol should be measured almost immediately after it is poured into the measurement beaker [72] and the temperature should be kept constant and monitored. Lastly, since alcohols are inflammable and have an acute inhalation toxicity, working with these liquids requires a safety protocol, such as the use of special fire-proof storage cabinets and handling under a fumehood [132]. 

#### 5.1.2. Saline

The dielectric properties of different concentrations of NaCl (saline) solutions at various temperatures have been modelled in the microwave frequency range [49,75,136,137,138]. Specifically, Stogryn provided models in the gigahertz range for computing the complex permittivity of saline as a function of temperature and concentration (between 0.25 M and 0.5 M) in order to allow these liquids to be used as references [136]. More recent models, based on extended experimental data, are now available for solutions having concentrations between 0.001 mol/L and 5 mol/L in the frequency range of 0.10–40 GHz, for any temperature between 0 °C and 60 °C [49,130,133,137,138]. Although alcohol models are, generally, more accurate than saline models, saline solutions are the most convenient reference liquids used [133].

Among all of the saline solutions, 0.1 M NaCl solution is the most commonly used reference liquid to assess the uncertainty in measuring the dielectric properties of biological materials, since it has similar dielectric properties to those of biological tissues [43,44,133]. Furthermore, 0.1 M NaCl is stable in temperature and electrical properties during storage and handling. At room temperature, saline does not evaporate quickly like alcohols. Saline solutions are also straightforward to prepare (hence, commercially-bought solutions are cost-effective) [133] and to use. Saline solutions are also less dangerous than alcohols and, thus, they do not require the use of fire-proof storage cabinets or handling under a fumehood. For 0.1 M NaCl, models that cover relatively wide frequency and temperature ranges are available [133]. However, saline may not be the best choice as a validation liquid when DI water is used as calibration, since these two liquids have very similar dielectric properties in the microwave range. Furthermore, due to poor traceability of the data used to obtain the models in [133] (since the data was acquired with only a single measurement system and a single measurement technique, and then compared to reference data measured under unknown conditions), the saline models are likely not as accurate as the models for alcohols. 

To this extent, future studies aimed at improving the reliability and accuracy of saline models have the potential to support dielectric data validation and uncertainty calculations. 

#### 5.1.3. Other Liquids

Several other liquids, such as formamide [75,84,134,137], DI water [94,124,132], dimethyl sulphoxide (DMSO) [94,132,139], and acetone [94,132,140], have been used as reference liquids. 

Formamide is a polar organic solvent, which has a relative permittivity of approximately 110 at low frequencies that drops down to a high-frequency value of around 7 [134] (when handled at room temperature). The temperature-dependent model for characterising the dielectric properties of formamide across the microwave frequency range was developed by Jordan et al. and, more recently, by Barthel et al. using waveguide interferometry [75,134,135]. The parameters of different models were found at discrete temperatures in the frequency range between 0.2 GHz and 89 GHz. The reliability of the model in Jordan et al. is affected by the limited discrete frequency points used in the dielectric measurements from which the model has been obtained [134]. In both Jordan et al. and Barthel et al., the dielectric models are available only for limited discrete temperatures [134,135]. Also, since formamide is toxic, a custom handling protocol is required.

When it is not used as the broadband load in the calibration procedure, DI water represents an advantageous validation liquid [117,124]. In fact, DI water has dispersive properties similar to those of biological tissues and has been accurately modelled in the microwave frequency range for any temperature between −4.1 °C and 60 °C [124]. DI water also has the advantage of being a stable liquid and does not require special handling. 

Dimethyl sulphoxide (DMSO) is a highly polar organic reagent that has a high relaxation frequency. DMSO has relative permittivity values similar to those of muscle tissues. Dielectric models for DMSO have been developed that cover a wide frequency range [139] and different temperatures [132]. DMSO is hygroscopic [94,132] and when it evaporates the liquid temperature increases, causing an increase in relative permittivity values [132]. Therefore, like with many alcohols, care should be taken to keep the liquid in a closed container as much as possible.

Acetone is a polar organic solvent that has intermediate permittivity values, which have been modelled only in the upper microwave frequency range [140]. Acetone requires special handling because it has a boiling point of 56 °C and has the potential to soften some plastics [94,132].

Liquid properties and information about available models and storage/handling procedure related to the most common categories of reference liquids are reported in Table 3. The column “Models” contains the most referenced models, i.e., those which cover the widest frequency range and largest, most continuous temperature interval.

### 5.2. Uncertainty Calculation

It is always good practice to report uncertainty along with measured values. However, in dielectric measurement studies, the definitions used for uncertainty, including how they are calculated and reported, have varied widely. 

Today, the uncertainty of measurements is generally calculated according to the guidelines defined by the National Institute of Standard and Technology (NIST) [43,44,75]. Multiple measurements performed on the same material of known dielectric properties enables determination of uncertainty of the measurement system in terms of the repeatability and accuracy. Considering the definition of uncertainty reported in [43,75], the repeatability of the measurement may be expressed quantitatively in terms of the characteristics (e.g., standard deviation) of data repeatedly acquired under the same measurement condition, as defined also in [74]; while the accuracy may be defined as the average percentage difference between the dielectric properties of the acquired data and those of the model [43,44]. These definitions represent practical methods of calculating these parameters. In this way, the repeatability varies between measurements and gives the extent of random errors, while the accuracy is constant across measurements. 

The uncertainties in repeatability and accuracy both contribute to the total uncertainty in the dielectric measurements [43,44,74,75]. For example, the combined standard uncertainty may be calculated as the root sum squared of the standard uncertainties [43,75]. In Peyman et al., the standard uncertainties associated with Type A errors (repeatability), Type B errors (in the calibration and measurement of the reference liquids), VNA drift, and cable variations, were estimated and included in the combined standard uncertainty calculation [43]. These uncertainties were determined for 0.1 M NaCl and, undoubtedly, tissue measurements will be impacted by more and/or different uncertainties. 

Alternatively, in Gregory et al., uncertainties associated with specific input parameters were thoroughly evaluated by means of Monte Carlo modelling [141]. Notably, this modelling technique also enables estimation of uncertainties in measurement scenarios when there are no suitable reference materials available (e.g., with similar material properties or frequency range) [141].

According to the NIST guidelines, the best practice for expressing uncertainty is to report the mean measured value along with a confidence interval (CI) of 95% [74]. For dielectric measurements, one may wish to present these parameters separately for both the real and imaginary parts of permittivity. 

In the next section, techniques related to minimisation or compensation of tissue-related confounders are described.

## 6. Tissue Sample Preparation and Measurement Procedure

Tissue-related confounders may be the major cause of measurement uncertainty, since the total combined uncertainty for measurements on liquids is relatively small compared to that of tissue measurements [43]. Uncertainties associated with measuring tissue properties seem to be primarily related to the complex structure of biological tissues [39,43,122].

In order to reduce tissue-related confounders, it is useful to plan each set of measurements according to the experimental goal. The first step involves the choice of the target animals (since their age or weight could affect the dielectric properties [43,122,142]) and the sample tissue type. Aside from the source species, the number of samples should be chosen based on the scientific question. The following steps include the analysis of the various tissue-related confounders and the evaluation of different methods that aim to reduce or compensate for these confounders. 

In the next subsection, the confounders related to probe choice, sample preparation, and handling are first described. Then, a discussion of the confounders that need to be considered during the measurement procedure is provided.

### 6.1. Probe Selection Considerations

Open-ended coaxial probes are suitable for use with materials that are liquid or semi-solid [95], homogeneous [95], have flat surfaces [66,95], and have a semi-infinite thickness [39,43,95]. Tissues are generally semi-solid (with the exception of bone), but they are not always homogeneous or have flat surfaces, and tissue samples that are much thicker and larger than the probe tip are not always easy to prepare. Hence, probe selection is affected by three main biological factors: Sample size, heterogeneity, and tissue surface. The desired frequency range of the measurement may also impact the choice of the probe.

#### 6.1.1. Sample Size, Sensing Volume, and Heterogeneity

Dielectric spectroscopy techniques permit the acquisition of the average complex permittivity of the interrogated volume. Thus, the probe should be selected such that the sensing volume only contains the tissue sample of interest and no other material. Since probes with a small diameter have smaller sensing volumes compared to large flanged probes, the sample size has to be taken into account and compared to the sensing volume of the probe [71,72,76]. 

The sensing volume may be evaluated by performing preliminary experiments with different combinations of materials. To this end, Meaney et al. analysed the dielectric property change in two-layer materials, consisting of saline or DI water with Teflon or acrylic, by varying the thickness of the liquid layer to determine the influence of materials at different depths on the measurement. The experimental results suggested that the dielectric properties are dominantly influenced by the material present within only the first 200–400 microns from the probe tip, and that this depth did not vary significantly across frequency or material properties [80]. This was a key finding as previous studies had assumed a much larger region on the order of several millimetres [11]. While Meaney et al. and Hagl et al. both investigated the depth into a tissue that contributes to the dielectric measurement, they defined the depth parameter differently [71,80]. More recently, Porter et al. demonstrated how different definitions of the sensing depth can impact the determined sensing depth value and highlighted that, for some definitions, the value does depend on the frequency and dielectric properties of the tissues occupying the sensing volume [83]. The work of Porter et al. also confirmed the findings of Meaney et al., in that the experimental results demonstrated that the tissue in contact with the probe has a greater impact on the measured dielectric properties than deeper tissues [82]. Nevertheless, because the sensing volume may be affected by the intrinsic dielectric properties of the investigated sample, further experiments involving the analysis of materials with more complex structures across both radial and axial directions are needed in order to define the sensing volume accurately for complex tissue samples.

Heterogeneity of biological samples is a further factor to consider when choosing a probe, since it is challenging to determine the tissue-specific dielectric properties in an extended heterogeneous volume interrogated by the probe [39,43,76]. To date, the impact of tissue heterogeneity with only simplified configurations has been thoroughly modelled. For example, in Chen et al., it was demonstrated that, for bilayer materials, the permittivity of either layer can be calculated from the reflection coefficient without the need for information on the thickness of the first layer or the probe capacitances [143]. Models for the effective dielectric properties of bilayer materials, in general and in particular for coaxial probes, have also been presented in [107]. These results were also extended to a general multilayer material scenario [107]. Furthermore, Huclova et al. used a numerical three layer skin model to examine how variations in the layer properties (including thickness and permittivity), impact the dielectric measurement across frequency [144]. More complex heterogeneities have yet to be thoroughly investigated or quantified. Specific challenges associated with heterogeneous tissues (aside from their impact on probe selection) are discussed in Section 7.

#### 6.1.2. Tissue Surface Characteristics

In addition to the sample size and heterogeneity, the quality of the tissue surface is another consideration when selecting the appropriate probe to use. Surface irregularities may contribute to inadequate probe-tissue contact and poor repeatability of dielectric measurements [42,43,60]. Characterisation of the tissue surface permits the identification of the tissue area or points that are most suitable for the acquisition of dielectric information [145]. For instance, thick samples and even surfaces are preferable to thin and uneven surfaces in order to ensure good probe contact with the tissue sample [42,60,95]. From the authors’ experience, the use of a smaller probe on uneven tissue surfaces results in more reliable measurements, especially if these areas are limited or spatially restricted. Lower uncertainty in the measurements from smaller probes on uneven surfaces may be attributed to smaller forces being applied on smaller surfaces. Indeed, large uneven surfaces require the application of higher forces (and, consequently, higher pressures) to prevent the presence of air gaps between the probe and the tissue. An increased probe-sample pressure may cause fluid accumulation at the probe tip [39,43] or tissue damage [95], both of which can affect the tissue dielectric properties and lead to inaccurate data. 

In summary, the probe should be selected not only on the basis of the probe characteristics and specifications (i.e., frequency range, permittivity range, temperature range, mechanical resistance) discussed in Section 3, but also based on the properties of the tissue under investigation. The size of the selected probe has to be consistent with the sample surface, size, and heterogeneity in order to achieve good probe-tissue contact and accurate measurements in a homogeneous region. 

After selecting the probe, but before measuring the dielectric properties, it is recommended to carefully plan the tissue preparation and handling procedures in order to reduce tissue-related confounders, such as sample cooling, dehydration, and damage.

### 6.2. Tissue Preparation and Handling

Tissue measurements can be performed in vivo or ex vivo; the tissue preparation and handling will be different in each case. Often, for reasons of convenience (i.e., patient safety, ethics) or due to difficulties in establishing a good probe-sample contact with in vivo tissues, dielectric measurements of animal and human tissues are performed ex vivo. 

#### 6.2.1. In Vivo vs. Ex Vivo Measurements

Several authors have reported on whether or not differences exist in tissue dielectric properties acquired in vivo and ex vivo. These works will be discussed here in chronological order. Initially, Burdette et al. performed in vivo measurements on canine muscle, kidney cortical tissue, and fat tissue, and differences were found between acquired in vivo data and reported ex vivo data [39]. In particular, for in vivo canine fat tissue, the measured permittivity values were a factor of approximately 1.5 to 3 times larger than the in vitro permittivity values acquired previously by other authors [39,52,146]. This difference in dielectric properties was most likely due to differences in water content, in temperature, or actual physiological differences between living and non-living tissues [39]. Next, Kraszewski et al. performed both in vivo and ex vivo dielectric measurements on rat and cat tissues, finding only dielectric changes less than the uncertainty at frequencies between 100 MHz and 8 GHz [40]. Schwartz observed that the permittivity and conductivity of frog heart, in the frequency range 0.2–8 GHz, were higher in vivo than ex vivo, with the difference being attributed to blood perfusion changes [41]. More recently, a variation between in vivo and ex vivo dielectric properties was found by Gabriel et al. and Peyman et al. in skin, spinal cord, skull, long bone, and bone marrow in the microwave frequency range [42,43,66,142]. Similar differences were not observed in other tissues, but might indicate unavoidable contamination of tissues with blood or other body fluids [43]. From the analysis of normal and malignant human liver tissues, O’Rourke et al. found a statistically significant difference between in vivo and ex vivo normal liver tissue, but not between in vivo and ex vivo malignant liver tissue [37]. Furthermore, Halter et al. evaluated the changes of breast cancer dielectric properties between in vivo and ex vivo measurements and found about a 30% drop in the magnitude of the permittivity in tissues analysed 300 min after excision [10]. More recently, Shahzad et al. found that over the 210 min following excision, the relative permittivity of liver tissue, as measured on the surface of the sample, decreased by 32 points [147]. However, this decrease was attributed fully to dehydration of the surface of the tissue sample as dielectric measurements conducted on the interior of the sample did not change considerably over the same time period [147]. The exact magnitude of the change in dielectric properties from time of excision to time of measurement, caused by dehydration and temperature effects, will vary based on the tissue type, the environment that the tissue is stored in, and the tissue handling conditions.

As is clear from the varied results of these studies, there is no consensus on: (i) Whether a difference in the dielectric properties of in vivo and ex vivo tissues exists over the microwave frequency range; and (ii) if a difference does exist, the magnitude and direction of it. Despite these results, the difference between in vivo and ex vivo data in the microwave frequency range is, generally, attributed to the temperature change and tissue dehydration [10,30,39,43,86], and recent studies following best practice in dealing with these confounders suggest no significant difference in the dielectric properties measured from in vivo and ex vivo measurements [84,148]. Therefore, following best measurement practice, it is advantageous to keep the temperature constant during dielectric measurement using a temperature controlled container or a water bath [29,40,43,44,122,127,142] and to minimise dehydration by limiting the time between excision and measurement to a few hours [27,30,40,42,43,62,65,76,78,86,127,142,149,150,151]. At frequencies lower than 100 MHz, a larger variation between in vivo and ex vivo properties is found. This difference is attributed to physiological parameters, such as blood flow in vessels [27,39,65,86,151], ischemia [10,86,150,151], heart rate [43], arterial pressure [43,86,150], respiration rate [43], and air content in lungs [149], which can affect the permittivity and conductivity values at these frequencies.

In the following subsections, the best-practice steps involved in both in vivo and ex vivo measurements are described: From surgical intervention, to sample access and excision, transportation, handling, and processing. In each step, all potential tissue-related confounders, as well as the different methods used in previous works to compensate for them, are reported.

#### 6.2.2. Surgical Intervention, Sample Access, and Excision

The first step in defining a sample handling procedure involves identifying the surgical methods to be used for tissue access and excision. It is necessary to define a surgical protocol that minimises tissue property modification. The main factors interfering with the dielectric acquisition concern the use of chemicals [39,127], which alter the body physiological condition, the use of tools or techniques [10], which may damage tissues, and the tissue exposure and cooling during the surgical operation [39,40,41,43,152].

It is useful to test for, and take into account, the effect of anaesthesia or other pharmaceuticals, which are used on animal/human tissues and physiological parameters. For instance, Burdette et al. observed a decrease in body temperature due to anaesthesia [39].

During the surgery, contact with the tissue should be minimised in order to avoid any damage or contamination. For human in vivo studies, the measurement tools need to be sterilised prior to surgery. Normally, steam sterilisation is performed prior to calibration [10,37] and a calibration refresh could be performed in the sterile environment before the in vivo measurements [10]. Furthermore, for in vivo measurements, the temperature tolerance of the probe (that depends on the probe fabrication materials) needs to be taken into account when selecting the sterilisation (or autoclave) procedure. For instance, steam sterilisation is, generally, performed at temperatures within 125 °C, while dry heat sterilisation can be conducted at temperatures up to 190 °C.

Other important confounders to take into consideration in the operating room during in vivo measurements are those related to the tissue exposure to air. Specifically, air contributes to tissue cooling (from body temperature to room temperature) and to tissue dehydration. Different techniques have been adopted in in vivo measurements to prevent tissue cooling and dehydration. For example, Ranck and BeMent performed experiments within a few minutes from the surgical cut used to expose the interior tissues, and used warm saline to wet the measurement region [152]. Schwartz et al. rinsed the tissues and kept them moist with frog physiological solution [41]. Hart and Dunfee applied Ringer’s solution with a medicine dropper to the muscle to prevent drying between the measurements [153]. However, these methods to reduce dehydration can impact the dielectric property measurement, since the solutions used have their own dielectric properties that will then contribute to the dielectric measurement of the tissue. Thus, the use of solutions, especially saline, should be avoided. More commonly, tissue dehydration during an in vivo measurement is minimised by reducing the time between the surgical cut performed to expose the tissue and the dielectric measurement, and covering the area of interest with another tissue between measurement times [39,40,43]. This technique does not alter the tissue properties and also minimises tissue cooling. The tissue temperature should be measured frequently, so that any temperature change is taken into account during data analysis. 

In previous works, the in vivo tissue temperature was monitored using thermocouple probes [27,29,62] and, more recently, fibre-optic thermometers [29,30]. Infrared thermometers may also be used for tissue temperature monitoring, since they are portable and do not require sample contact [79]. The same sensors can also be used in ex vivo measurements. A further crucial point in in vivo measurements concerns the probe positioning. Typically, in ex vivo scenarios, the probe–tissue contact can be verified by visual inspection; however, this approach can be challenging in a surgical setting. The probe positioning cannot be accurately planned prior to surgery; thus, it is normally decided in the surgical theatre. 

#### 6.2.3. Tissue Transportation

When ex vivo measurements are performed, the excised sample may be transported from the operating theatre to a secondary location for measurement, characterisation, or histology (details on histological analysis are presented in Section 7). The time between excision and ex vivo measurements is minimised to prevent tissue dehydration [27,30,40,42,43,62,78,86]. Aside from water content change, care should be taken during tissue transportation to avoid changes in the sample temperature. Since the temperature has a systematic impact on the measured dielectric spectrum of biological tissues, it is usually necessary to transport the tissue in hermetically-sealed, temperature-controlled containers [29,44,76,142]. 

#### 6.2.4. Tissue Handling

In order to prevent tissue contamination, dehydration, and damage, sample handling prior to the ex vivo measurements should be minimised [39,71,76,142]. The sample temperature can be kept constant during the measurements using a water bath [29,40,43,122,142]. As the temperature setting of the water bath may not be equivalent to the tissue temperature, the tissue temperature should still be verified using an infrared or fibre-optic thermometer [29,30]. In this way, the tissue temperature variation can be taken into account during data analysis. Details on how tissue temperature affects the measured dielectric properties are reported in Section 6.3.3.

If the tissue sample is to be analysed histologically, the measurement points should be marked. Sample marking is necessary to ensure that the histological analysis involves the portion of tissue corresponding to the volume interrogated by the probe. Thus, a good correspondence between the tissue histological and dielectric properties can be found. Further details about the histological characterisation of tissue samples are reported in Section 7. In previous works, acrylic ink [76,79] or pins [10] have been used as sample markers. When ex vivo measurements are performed at the same locations where in vivo measurements were taken, it would be wise to test the effect of the marker on tissue dielectric properties before experimental implementation in order to prevent tissue modification or damage by the marker. Lastly, in order to maintain the integrity of the tissue, the use of additive and preservatives should be avoided until the measurement is completed [127].

Having presented the confounders that should be considered during the planning of the tissue measurement procedure, in the next subsection the actual measurement procedure and the key confounders that affect tissue dielectric property measurements are discussed.

### 6.3. Procedure for Tissue Measurements

After the equipment set-up, calibration, and validation, the measurements on in vivo or excised tissues can be performed. It is important to note that some confounders cannot be minimised even with careful preplanning. These confounders need to be controlled, monitored, or compensated for during the measurement phase. In order to minimise the effects of the environmental parameters on tissue dielectric properties, it is advantageous to perform measurements in a climate (temperature, pressure, and humidity) controlled room [43,127].

In the following paragraphs the main confounders occurring during the measurement phase, such as measurement region choice, probe-tissue contact, and pressure, as well as tissue sample temperature, are discussed. 

#### 6.3.1. Measurement Region Choice Confounders

The confounders mentioned in Section 6.1 (i.e., probe sensing volume, tissue thickness, tissue surface, and sample heterogeneity) need not only be considered in the planning phase, but also need to be controlled and managed in relation to the choice of the measurement region. Additional considerations may also be needed, for instance, in order to prevent undesirable reflections negatively affecting the measured data, Abdilla et al. placed a shorting block under the sample to check for any reflections from the sample boundaries [44]. 

Confounders intrinsic to the tissue type include: Fibre orientation in anisotropic tissues, presence of blood vessels, and high heterogeneity. It was observed that anisotropic tissues, such as muscles, present different dielectric properties according to the measurement directions along or across the fibre. Specifically, it has been found that in the microwave frequency range (from 200 MHz to 20 GHz) the permittivity values between the two sets of measurements are not substantially different. On the other hand, at lower frequencies (10^−5^–1 MHz) the fibre direction can change the relative permittivity by 100% [42]. Blood vessels are non-uniformly distributed in tissues and may make up roughly 30% of their volume [144], so the probe position relative to that of blood vessels should be checked by visual inspection [65,151]. In highly heterogeneous and mechanically stiff tissues the uncertainty is generally higher and, in order to minimise the random errors arising from tissue heterogeneity and complexity, it is useful to repeat the measurements at multiple points [43,44,75]. For instance, Peyman et al. stated that as many measurements as possible should be taken on each sample tissue and, in her study conducted in 2005, at least six measurements were taken on each tissue [43]. In most other dielectric studies, three to five measurement locations were, generally, selected on each tissue sample [27,40,44].

#### 6.3.2. Probe-Tissue Contact

Having selected the most suitable measurement region, the probe is placed in contact with the sample. From the authors’ experience, in order to reduce the uncertainty due to probe and cable movement, in both ex vivo and in vivo measurements (in in vivo measurements only when the animal size is relatively small), it is convenient to move the sample towards the probe using a lift table until the entire probe aperture makes firm contact with the tissue sample as opposed to moving the probe during the measurement procedure. 

Measured reflection coefficient data is extremely sensitive to the probe positioning relative to the sample surface. A high variability in the dielectric properties can be attributed to variability in probe-tissue contact. Thus, a firm contact between the probe and the tissue [76,93] is key. A good quality contact reduces the impact of confounders that increase the measurement uncertainty, such as pressure differences [39,43,80,97,149], air gaps [70,93,95,126], and biological fluid accumulation at the probe tip [39,43]. In most works, these factors have been monitored by a close visual inspection [29,41,43,76,95]. In order to keep the applied pressure constant in ex vivo measurements, weighing scales or force sensors can be placed underneath the sample holder [79]. In fact, the application of a steady pressure contributes to more repeatable measurements [39]. However, in the literature to date, there is no work that quantifies the error in the measured data in terms of the variation of the applied pressure. The authors have performed a number of experiments to quantify the error introduced by probe pressure variations, but observed that the outcome found for one measurement point could not be extended to all the measurement points across the sample. For instance, within the same tissue sample, there can be some differences in terms of sample thickness, tissue mechanical properties, water content, and surface irregularities, which may require the application of different probe pressures on the same sample. Thus, no specific, fixed pressure can be reported for all samples. However, a technique that may be used to obtain a good quality contact is as follows. First, a low pressure is applied to the probe to contact the sample. This low pressure, if too low, can lead to data inconsistencies when repeated measurements are taken at the same point (due to air gaps). If this occurs, a pressure adjustment can be undertaken until measurements at the same location are repeatable. Conversely, the application of high pressure, if too high, can cause tissue compression and can prompt fluid from within the tissue to rise to the tissue surface, or worse, can cause tissue damage [127,149]. In previous works, sample contamination by biological fluids has been reduced by using cotton wipes/swabs [43,99,127,142,152] or suction [43]. However, it should be noted that the suction method is more invasive and has the potential to dehydrate the sample.

#### 6.3.3. Temperature Effects

During dielectric measurements, as discussed in Section 6.2.1, the temperature needs to be controlled and monitored. While different techniques used to monitor or control the temperature have been discussed in earlier sections, in this subsection the effect of temperature on tissue dielectric properties is examined. 

In previous studies, the dielectric properties of biological tissues at discrete frequencies and temperatures were measured and, for small temperature variations, they were presented in terms of linear temperature coefficients, which are defined as the percent change in either permittivity or conductivity per degree Celsius [53]. The provided linear temperature coefficients are limited to a number of specific discrete frequencies and temperatures [27,30,62]. Outside of these frequencies and temperatures the impact of temperature on the dielectric properties may no longer be linear [30]. A brief summary of the previously published temperature-dependent dielectric properties data is presented in Lazebnik et al. [30]. In the microwave frequency range, the change in relative permittivity is, at most, 2% per degree Celsius and the change in conductivity is between 1% and 2% per degree Celsius, depending on the tissue and on the frequency and temperature range considered. Generally, the relative permittivity and conductivity trends with temperature differ over frequency. However, the magnitude change in both permittivity and conductivity per degree Celsius tends to be higher at lower frequencies in most biological tissues [27,30,62]. Lazebnik et al. developed a model to characterise the temperature-dependence of liver tissue dielectric properties over the microwave frequency range [30]. In particular, from the liver dielectric measurements, Lazebnik et al. identified different “cross-over” points in the trends of both relative permittivity and conductivity with temperature. In relative permittivity, the cross-over point was found at about 4 GHz. Below the cross-over point, the permittivity decreases slowly as temperature increases and, above the cross-over point, the permittivity increases with temperature. For conductivity, two cross-over points were found: One near 2–3 GHz and the other near 16 GHz. Below the first cross-over point, the conductivity increases slowly as temperature increases. Between the two cross-over points, the trend reverses, and above the second cross-over point, the conductivity again increases as temperature increases. The same trends were also found for water [30]. 

More recently, temperature coefficients were provided for a wider temperature range (up to 100 °C) at the discrete frequencies of 915 MHz and 2.45 GHz, which are of interest for microwave liver tissue ablation [31,154]. Brace et al. found that linear temperature coefficients across the 5–50 °C range agreed well with the results of Lazebnik et al., with coefficients of −0.22 and −0.18 in relative permittivity for the two frequency points, respectively, and coefficients of 1.29 and −0.2 for conductivity [31]. From 50 °C to 100 °C, both relative permittivity and conductivity were found to decrease by as much as 50%, due to both irreversible damage of the tissues and tissue dehydration [31]. In summary, the temperature coefficients for both permittivity and conductivity depend on tissue-type, on frequency, and on the considered temperature range. Knowledge of these temperature coefficients can be used to compensate for the effect of the temperature change during tissue dielectric measurements. 

**Figure 2:**
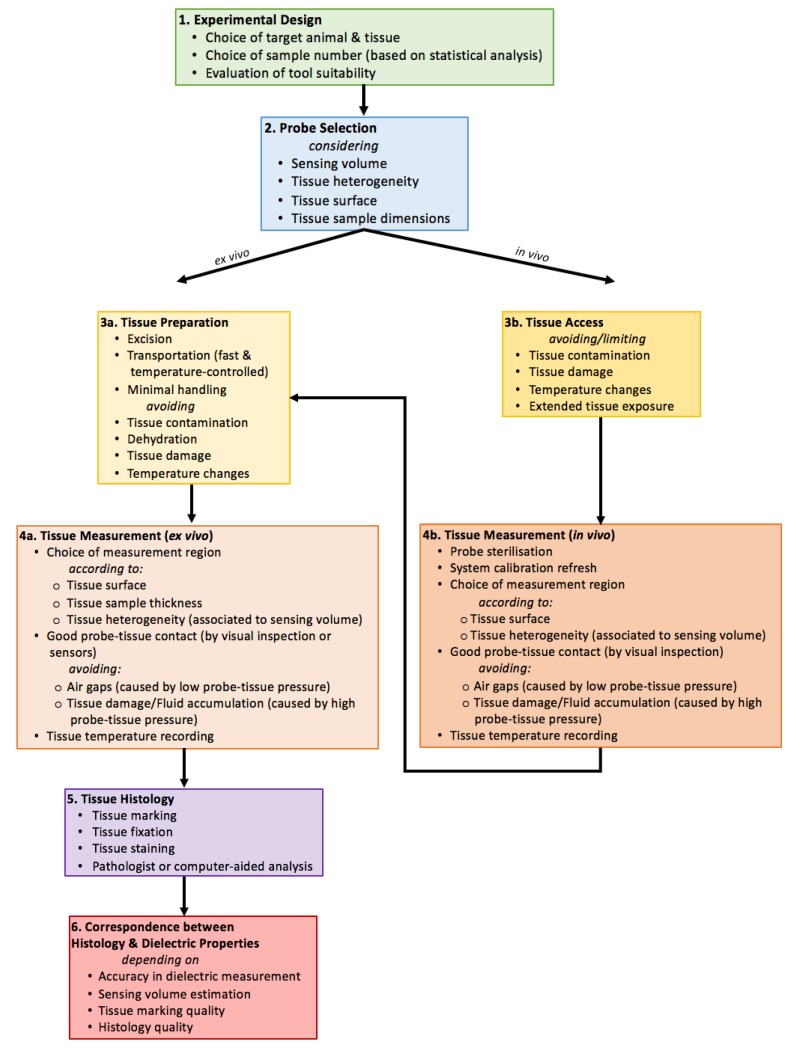
Flow chart of the common steps to minimise tissue-related errors in in vivo and ex vivo measurements.

In this section, the importance of preplanning the measurement procedure was highlighted, the measurement process overviewed, and the main confounders involved in the measurement were described. The most common practices adopted to minimise tissue-related errors are summarised in Figure 2. In the next section, histological analysis of tissue samples is discussed as a method to reduce the confounders related to the intrinsic heterogeneity of biological tissues.

## 7. Tissue Sample Histological Analysis

Histology is the study of the microscopic structure of cells and tissues; while histopathology refers to the same, but with diseased tissue [155,156]. There are multiple steps involved in the histological analysis of a tissue sample: The sample must be fixed, embedded in wax, sliced, mounted on slides, stained, and then imaged [157]. Following these steps, the slices are ready to be analysed by a pathologist. The pathologist is able to examine the images and determine: (i) The types of tissues present; (ii) if diseased tissue is present, the disease grade and other characteristics (for example, with breast cancer, the hormone receptor status) [156]; and (iii) the distribution of the tissue types within the sample. Histological analysis is, especially, required after the acquisition of the dielectric properties of a heterogeneous tissue sample in order to determine the tissue types present in the sample and their relative spatial distribution. This is important because the dielectric properties of a sample are determined by those of its constituent tissue types; thus, the histological analysis enables the attribution of measured dielectric properties to the appropriate tissue type.

Many studies performed in the literature involve only homogeneous (or assumed homogeneous) tissues and, thus, the samples do not undergo histological analysis (for example, liver tissue [27,44]). In this section, the focus is on heterogeneous tissue samples. Measuring the dielectric properties of heterogeneous tissues is inherently challenging as spectroscopy has the effect of averaging the dielectric properties throughout the sensing volume that is illuminated by the electromagnetic field [11]. Thus, in the next subsection, confounders that can contribute to any histological analysis are detailed, along with ones that are of specific concern for dielectric measurements of heterogeneous tissues. Finally, histological analysis methods used for attributing dielectric properties to heterogeneous tissues from the literature are overviewed, and the best practice techniques that are known are highlighted. 

### 7.1. Factors Impacting Histological Analysis

The procedures involved with histological preparation of the tissue are applied by pathologists thousands, if not millions, of times per year. In fact, there are more than 14 pathology tests examined per person in the UK each year, and pathological analysis is a part of 70% of all diagnoses [158]. However, the methods are not without flaws. In particular, poor fixation of the sample can lead to changes in the tissue structure [11,157] and uneven levels of staining can result in images that are incomplete or out of focus [157]. Slide digitation can have variations in lighting conditions and magnification that can affect interpretation of the results, particularly when comparing across slices [157]. Each of these issues increases the challenge of interpreting the dielectric measurement of heterogeneous samples based on the histology of tissues samples and makes it especially difficult to compare between studies. Furthermore, the histological interpretation of a slice itself is subjective and variability in results between pathologists are possible [159,160,161]. Computer-aided diagnosis (CAD) and prognosis (CAP) methods are currently being investigated to create a fully automated analysis that is faster and more consistent than a human-based analysis [157]. An excellent review of challenges associated with histopathological analysis can be found in Veta et al. [157].

### 7.2. The Link between Heterogeneity, Histology, and Sensing Volume 

When performing histology to support interpretation of the tissue content contributing to a dielectric measurement, it is important to include in the histological analysis all of the tissues that are within the sensing volume. However, at the same time, the histological analysis should not include any tissues that are outside of the sensing volume. In this way, only, and exactly, the tissues that have contributed to the measurement are analysed.

**Figure 3:**
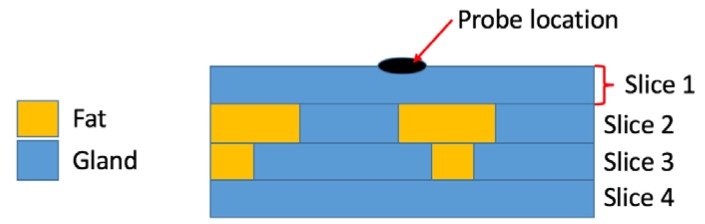
Diagram of sample composition by tissue type (fat—orange, gland—blue). A side view of the sample is shown, with slices marked. The dielectric probe measurement location is denoted with a black oval on the top of Slice 1.

As an example, Figure 3 demonstrates the importance of matching the sensing depth with the number and thickness of slices taken into consideration in the histological analysis. If only Slice 1 is analysed, the tissue is found to be composed entirely of homogeneous glandular tissue. If the sensing depth is equal to the thickness of Slice 1, then the measured dielectric properties will be entirely the result of this layer of homogeneous gland tissue. Alternatively, if the sensing depth is equal to, say, the combined thickness of Slices 1 and 2, then the total sensing depth region is occupied by 25% fat tissue and 75% glandular tissue (as Slice 1 is 100% gland, and Slice 2 is 50% gland and 50% fat). Both of these tissue types will contribute to the measured dielectric properties. However, the contribution is not proportional to the tissue type representation (i.e., 25% fat and 75% gland) as the layer closest to the probe has the dominant effect [11,80,81]. Furthermore, not all of a given tissue is occupied fully by cells of that tissue type [12], thus, an additional layer of complication comes into the example based on how to determine what regions are actually “fat” and which are “gland”. Obviously, as more slices are involved in the analysis and the tissue becomes more heterogeneous, the more challenging it becomes to conclusively determine the tissue composition breakdown. It is also important to re-emphasise here that the sensing volume is dependent on the tissue content (namely, the tissue dielectric properties), so, ideally, the change in the sensing volume should be taken into account on a sample-to-sample basis, as discussed in Section 6. 

### 7.3. Histological Analysis Techniques in Dielectric Studies

A limited number of works involving histological analysis for attributing the measured dielectric properties of heterogeneous tissues have been presented in the literature. Of these, some use pathology to categorise tissue samples by type [79,86], while a select few process the tissue for microscopic analysis [10,11,12,76]. In general, histology for dielectric characterisation is an area that requires further investigation [43]. The most common strategy is to obtain an average estimate of the tissue types present in the sample below the probe [10,11,76]. However, most recently a more quantitative method of counting each cell and corresponding the proportion of tissue with the measured properties has been proposed [12]. These techniques are described and compared in this section. For dielectric property measurements of heterogeneous tissue, breast tissues are by far the most common that have been analysed due to the need for these properties in medical microwave imaging of the breast. As a result, all pathology techniques discussed in this section have all been performed on breast tissues. 

In Lazebnik et al., several hundred dielectric measurements were taken from normal and malignant excised breast tissue samples using an open-ended coaxial probe [11,76]. The measurement sites were marked on the tissue samples using a spot of black ink. The authors conducted a histological analysis of each sample based on the tissue composition inside the region of the sensing volume of the probe (3 mm deep × 7 mm across, for this measurement scenario, as determined in Hagl et al. [71]). In this way, a cross-section of each tissue sample was taken directly below the measurement location (i.e., the ink spot). Digital microscopy images were obtained and visually inspected. The tissue composition within the sensing volume was quantified based on the percentage of each tissue type residing within the slice under consideration. The two-dimensional cross-section was used to obtain an estimate of the tissue composition in the full three-dimensional sensing volume. The percentages of each tissue type (adipose (fat), glandular and fibroconnective tissue, along with benign and malignant tissue) were estimated visually by qualified pathologists [11,76]. A Kappa statistic was used to confirm consistency in the analysis between different pathologists. Several exclusion criteria were applied during the histological process. In particular, samples were eliminated from further consideration if the ink spot was not visible, if the ink had leaked into the tissue, or if the cross-sectional slice was deformed. In this study, nearly half of all samples (49.8%) were excluded based on difficulties during the histological procedure [76].

Following the studies by Lazebnik et al., Halter et al. performed a study that also examined the region under the probe using histological analysis. In Halter et al., the dielectric properties of in vivo and ex vivo breast tissues were measured in the microwave frequency range with open-ended coaxial probes [10]. After the in vivo tissue measurement was recorded, a biopsy clip was embedded in the tissue at the measurement location. The tissue was then excised and sectioned into 5 mm thick pieces. The excised samples were measured again (at the same site as for in vivo, as identified by the clip). Initially, the pathologist examined a 1 cm × 1 cm square area around the measurement location and, thus, the tissue types were estimated based on a large area. Later, the strategy was improved by inserting two pins covered in ink into the tissue on either side of the depression left by the probe in order to mark the measurement location. The tissue sample was fixed with formalin, stained, and then slides were prepared. The pin holes were then used during the analysis to determine the probed region in which the tissue types were estimated by the pathologist. In this study, details were not provided regarding whether or not samples had to be excluded from consideration due to histological challenges. The pathologist examined the tissue histology within the ~1 cm × 1 cm region, which was a horizontal slice relative to the probe position (i.e., perpendicular to the plane of the probe axis), unlike the vertical (or parallel) slice used in Lazebnik et al. However, in both cases, the full tissue composition within the sensing volume was estimated based on the given slice. Furthermore, as only one pathologist was involved in the study, a Kappa analysis similar to that in the study by Lazebnik et al. was not needed.

Most recently, in Sugitani et al., excised breast tissue samples were obtained and their complex permittivities measured using an open-ended coaxial probe [12]. The samples contained a combination of tumour tissue, normal fat tissue, and normal stroma (connective) tissue. The work aimed to calculate the effective permittivity of the tumour tissue based on the idea that each sample is an inhomogeneous mixture of cells with different permittivities. It was proposed, and confirmed, that, since the “tumour” tissue is composed of cancer cells mixed in with normal cells, the volume fraction of cancer cells in a sample affects the dielectric properties. In particular, the sample was treated with a hematoxylin-eosin stain and then digital images of each slice of the sample were taken. The slide images were analysed by counting the number of pixels of cancer cells and cells of other tissue types presented. The ratios of each type of tissue cell, relative to all of the cells in the slice, were calculated. The three-dimensional fractional volume of each cell type was calculated based on the two-dimensional slice using Bruggeman’s effective medium approximation theory [162]. This method has the advantage of being highly quantifiable—each cell is counted—however, the process is tedious and time-consuming. The work does not mention if any samples had to be discarded or were contaminated during the histological procedures. Furthermore, the sample analysis was not restricted to a specific sensing depth region (sample sizes ranged from 5 cm to 30 cm). A similar study on various types of malignancies was presented in Sugitani et al. [85], for which the pathological procedures were the same as those in Sugitani et al. [12].

Overall, there is no consensus in the literature to date on the best practice for conducting histology in relation to dielectric measurements. Furthermore, there has been no reported comparison of the different histology techniques used in the above-mentioned works, therefore, it is not known if some methods are more accurate than others. However, it is likely that some features from each of the studies lend themselves to obtaining more accurate data, for example, involving multiple pathologists and using Kappa analysis to verify consistency between them (as in the study by Lazebnik et al.) could only add to the study quality.

## 8. Discussion and Conclusions

Although notable progress has been made in achieving accurate dielectric data, the coaxial probe design still represents a limit for certain types of dielectric experiments. An improved probe design that could allow measurements over a wider spectrum of frequencies and across multiple tissue samples would be useful for future studies. Moreover, during the measurement procedure, the use of appropriate force and position sensors could considerably increase the stability of the measurement system and reduce tissue-related confounders that are strictly dependent on the expertise of the operator conducting the dielectric measurement (i.e., probe-tissue contact and probe-tissue pressure). 

Furthermore, interpretation of dielectric data acquired with the open-ended coaxial probe can be improved by quantitatively examining and compensating for tissue-related confounders that cannot be fully eliminated during the measurement procedure. To this extent, dielectric studies have modelled the effect of temperature, tissue dehydration, and animal age on the dielectric measurement of tissues. The quantitative characterisation of tissue-related confounders improves the interpretation of the acquired data and could support the interpretation of dielectric data from historic studies that did not provide information on all confounders. In order to clarify how such a characterisation could be done, a series of examples demonstrating how to determine the total uncertainty in a dielectric measurement are provided below.

This example scenario considers the case of dielectric measurement of mouse liver in the microwave frequency range, as the effect of the time from excision, temperature, and age of the mouse have all been quantified on liver tissue at these frequencies. In this example, it is assumed that the confounders of time from excision (TFE), temperature (T), and age (A), are the only ones impacting the dielectric data. The uncertainties introduced by these confounders are denoted as μ_TFE_, μ_T_, and μ_A_, respectively. The relative permittivity of liver has been acquired at room temperature, 3.5 h from excision from a 70 day old mouse. From the literature it is known that at the frequency of 900 MHz, the relative permittivity changes by 0.13% per degree Celsius [30], decreases by about 25% after 3.5 h from excision [147], and decreases by approximately 15% within 70 days of life [142]. This quantitative information needs to be taken into account for the calculation of the combined standard uncertainty according to the NIST guidelines [74,75], which provides μ, the total uncertainty added to dielectric data. A series of hypothetical studies are listed in Table 4, along with the resulting uncertainty. The technique of calculating combined standard uncertainty to achieve a total estimate on the uncertainty introduced in dielectric measurement studies due to tissue-related confounders can and should be applied to all datasets, which lack quantitative information on confounders. 

Given the importance of modelling the effect of the confounders for the interpretation and comparison of existing dielectric datasets, further investigation is needed to quantitatively examine the main tissue-related confounders (i.e., temperature, dehydration) on other tissue types and to analyse confounders not yet quantified (i.e., heterogeneity, probe pressure). Such quantitative analysis will not only improve the analysis of new dielectric data, but will also support the interpretation of historical dielectric datasets. 

In conclusion, this work has presented the dielectric measurement process with an open-ended coaxial probe and reviewed the most relevant works, with a critical discussion of known equipment- and tissue-related confounders. This work supports the aim of achieving accurate dielectric measurements of biological tissues. As these properties are fundamental to electromagnetic safety studies and medical technology design and improvement, an understanding of the measurement process is of interest to a wide ranging community of scientists and medical professionals.

## Figures and Tables

**Table 1:** Use of the commercial probe in recent works. Studies involving breast tissues are shaded in grey. The others involve liver tissues, apart from the porcine skin study in Karacolak et al. [116]*.* In the column “Relative permittivity range”, the extreme values in relative permittivity are reported from lower to higher frequencies.

Recent Works	Probe	Frequency [GHz]	Tissue Type	Sample Size	Relative Permittivity Range	Conductivity Range [S/m]
Halter et al. (2009) [10]	Slim form with 2.2 mm diameter (in vivo)	0.1–8.5	Ex vivo and in vivo	5 mm thick	In vivo breast tissue: 95–45	In vivo breast tissue: 0.1–10
	High temperature with 19 mm flange (ex vivo)		Breast tumour (human)		Ex vivo breast tissue: 50–35	Ex vivo breast tissue: 0.1–8
Karacolak et al. (2012) [116]	High temperature with 19 mm flange	0.3–3	Ex vivo skin (porcine)	45 × 45 × 4 mm^3^	50–36	0.4–2.2
Lopresto et al. (2012) [29]	Slim form with 2.2 mm diameter	2.45	Ex vivo liver tissue (bovine)	20 × 20 × 50 mm^3^	44.98–26.11 (temperature incremented from 15 °C to 98.9 °C, then decremented to 39.6 °C)	1.79–1.19 (temperature incremented from 15 °C to 98.9 °C, then decremented to 39.6 °C)
Sabouni et al. (2013) [86]	Performance with 9.5 mm diameter	0.5–20	Ex vivo breast tissue (human)	N/A	Breast tissue: 63–35	Breast tissue: 0.2–32
					Fibroglandular breast tissue: 40–20	Fibroglandular breast tissue: 0.2–16.3
Abdilla et al. (2013) [44]	Slim form with 2.2 mm diameter	0.5–50	Ex vivo muscle and liver (bovine, porcine)	60 × 60 × 40 mm^3^	Muscle tissue: 58–18 Liver tissue: 51–15	N/A(Loss factor for muscle/liver tissue: 32–10)
Sugitani et al. (2014) [12]	Slim form with 2.2 mm diameter	0.5–20	Ex vivo breast tumour (human)	50–300 mm diameter	Breast tumour tissue: 65–22	Breast tumour tissue: 0.1–25
					Breast fibroglandular tissue: 40–18	Breast fibroglandular tissue: 0.1–12
					Breast fat tissue: 12–6	Breast fat tissue: 0.1–3
Peyman et al. (2015) [84]	Slim form with 2.2 mm diameter	0.1–5	Ex vivo liver tissue (human)	20 mm thick	Liver normal tissue: 68–43 Liver tumour tissue: 68–32	Liver normal/tumour tissue: 0.7–5
Martellosio et al. (2017) [79]	Slim form with 2.2 mm diameter	0.5–50	Ex vivo breast tumour (human)	6 mm thick volume between 700 mm^3^ and 1500 mm^3^	Breast normal tissue: 64–3 Breast tumour tissue: 69–9	N/A (Breast normal tissue imaginary part: 41–0.1; Breast tumour tissue imaginary part: 45–4

**Table 2:** The standard calibration process: Common errors or confounders that occur for each step in the calibration process, along with the possible correction or compensation techniques. The open circuit, short circuit, and a liquid load material are shown as the three calibration standards.

Calibration Steps	Error or Confounder	Action for Correction or Compensation
**Equipment set-up**	Environmental parameter change [95] Probe contamination [27,37,39,121] Imperfect connection [39] Cable movement [43,76,80,115]	Control environmental parameters [113,122] Inspect and clean probe [29,41,42,43] Check connections [39] Fixing cable position (if not phase-stable) [29,44,86]
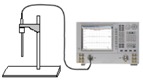 **Open**	Particles on probe tip [95]	Cleaning probe [29,41,42,43] Checking the Smith Chart [123] to ensure open-circuit impedance is being measured
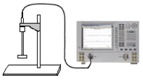 **Short**	Poor probe-short block contact [95]	Cleaning short block and probe [95] Reposition or re-contact short block with probe [95] Checking the Smith chart [123] to ensure short-circuit impedance is being measured
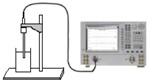 **Load**	Accuracy of liquid model [94,117] Liquid temperature [43,94,124,125] Air bubbles [48,71,126] Liquid contamination [43] Probe position in liquid [71]	Deionised water model has best accuracy [117] Monitor or control temperature [29,43,44,95,121] Re-immerse probe in liquid [95] Limit exposure to air [43] Place probe distant from beaker sides [71]

**Table 3:** Reference liquid properties, available models, and storage and handling procedures (where f = frequency, T = temperature).

Liquid	Models	Storage and Handling
**Methanol** (alcohol with intermediate permittivity values similar to breast tissue)	Debye model [132]: f = 0.1–5 GHz T = [10 °C, 50 °C], 5 °C increments	Inflammable and acute inhalation toxicity. Fire-proof storage cabinets required. Handling in fumehood required.
	Cole-Cole model [134]: f = 0.01–70 GHz T = [10 °C, 40 °C], 10 °C increments	Rapid evaporation may occur and should be avoided.
**Ethanediol** (alcohol with high permittivity values similar to breast glandular tissue)	Cole-Davidson model [132]: f = 0.1–5 GHz T = [10 °C, 50 °C], 5 °C increments	Inflammable and acute inhalation toxicity. Fire-proof storage cabinets required. Handling in fumehood required. Ethanediol is hygroscopic and when it evaporates the liquid temperature increases, causing an increase in relative permittivity [132].
**Ethanol** (alcohol with intermediate permittivity values similar to breast tissue)	Debye-Γ model [132]: f = 0.1–5 GHz T = [10 °C, 5 0°C], 5°C increments	Inflammable and acute inhalation toxicity. Fire-proof storage cabinets required. Handling in fumehood required.
**Butanol** (alcohol with low permittivity values similar to fat tissue)	Double Debye model [132]: f = 0.1–5 GHz T = [10°C, 40°C], 5 °C increments	Inflammable and acute inhalation toxicity. Fire-proof storage cabinets required. Handling in fumehood required.
**Saline (NaCl)** (polar liquid having dielectric properties similar to biological tissues)	Cole-Cole model [133]: Concentrations = [0.001 mol/l, 5 mol/l] f = 0.13–20 GHz T = [5 °C, 35 °C] (any intermediate T)	Storage in sealed containers. No special handling required.
	Cole-Davidson model [49]: Concentrations = [0.001 mol/l, 1 mol/l] f = 0.1–40 GHz T = 17 temperatures in the interval [10 °C, 60 °C]: 10 °C, 20 °C, increments of 2 °C in [24 °C, 50 °C], and 60 °C.	
**Formamide** (polar organic solvent having wide permittivity spectrum at microwave frequencies)	Cole-Davidson model [135]: f = 0.2–89 GHz T = [10 °C, 25 °C], 5 °C increments T = [25 °C, 65 °C], 10 °C increments	Toxic through inhalation, oral, or skin exposure. Fire-proof storage cabinets required. Handling in fumehood required.
**DI water** (polar liquid having well-known modelled properties)	Debye model [124]: f = 1.1–57 GHz T = [−4.1 °C, 60 °C] (any intermediate T)	Storage in sealed containers. No special handling required.
**Dimethyl sulphoxide (DMSO)** (highly polar organic reagent having high permittivity)	Debye model [132]: f = 0.1–5 GHz T = [10 °C, 50 °C], 5 °C increments Cole-Davidson model [139]: f = 0.001–40 GHz T = 25 °C	DMSO is exceptionally hygroscopic and needs to be measured as soon as the container is opened [132].
**Acetone** (polar organic solvent having intermediate permittivity values)	Static permittivity (since acetone has very high relaxation frequency) [132]: f = 0.1–5 GHz T = [10 °C, 50 °C], 5 °C increments	Acetone boiling point is at 56 °C [132].
	Budo model/confined rotator models [140]: f = 50–310 GHz T = 20 °C	Special handling is required, since it is a powerful liquid able to soften some plastics [94] and it is inflammable.

**Table 4:** Example calculations of total uncertainty in dielectric data resulting from tissue-related confounders under different measurement scenarios: Uncertainty due to time from excision (μ_TFE_), due to temperature (μ_T_), and due to age (μ_A_). μ is the total uncertainty added to dielectric data, calculated as combined standard uncertainty. Uncertainty data is for the relative permittivity of mouse liver at 900 MHz, obtained from the literature. Note that 0.91% is 0.13%/°C ∙ 7°C.

Case Scenarios	μ_T_	μ_TFE_	μ_A_	μ
Known TFE, Known age, Unknown T (between 18 °C and 25 °C)	0.91%	N/A	N/A	0.91%
Known T, Known age, Unknown TFE (within 3.5 h)	N/A	25%	N/A	25%
Known T, Known TFE, Unknown age (within 70 days old)	N/A	N/A	15%	15%
Known T, Unknown TFE (within 3.5 h), Unknown age (within 70 days old)	N/A	25%	15%	29.15%
Known TFE, Unknown age (within 70 days old), Unknown T (between 18 °C and 25 °C)	0.91%	N/A	15%	15.02%
Unknown TFE (within 3.5 h), Unknown age (within 70 days old), Unknown T (between 18 °C and 25 °C)	0.91%	25%	15%	29.17%
